# Electroacupuncture reactivates estrogen receptors to restore the neuroprotective effect of estrogen against cerebral ischemic stroke in long‐term ovariectomized rats

**DOI:** 10.1002/brb3.2316

**Published:** 2021-09-02

**Authors:** Yulong Ma, Erlong Niu, Fei Xie, Min Liu, Miao Sun, Ye Peng, Hang Guo

**Affiliations:** ^1^ Department of Anesthesiology The First Medical Center of Chinese PLA General Hospital Beijing China; ^2^ Department of Orthopedics 305 Hospital of PLA Beijing China; ^3^ Department of Pulmonary and Critical Care Medicine Chinese PLA General Hospital Beijing China; ^4^ Department of Orthopaedics Air Force Medical Center, PLA Beijing China; ^5^ Department of Anesthesiology The Seventh Medical Center of Chinese PLA General Hospital Beijing China

**Keywords:** critical period, electroacupuncture, estrogen receptor, estrogen replacement therapy, neuroprotection, stroke

## Abstract

**Background:**

Stroke is a sexually dimorphic disease and a leading cause of death and disability. Estrogen replacement therapy (ERT) confers beneficial neuroprotective effects if administered within a widely accepted time window called the “critical period.” However, very few studies have explored the idea of modulating the critical period to enable long‐term post‐menopausal women to regain more benefits from estrogen therapy. Here, motivated by previous findings that electroacupuncture could both alter estrogen metabolism and induce significant tolerance against stroke, it was explored whether EA could restore estrogen's neuroprotection against cerebral ischemia in long‐term ovariectomized (OVX) rats.

**Methods:**

We implemented 1 week(w)‐EA pretreatment on OVX‐10w or OVX‐20w rats, and tested the expression of estrogen receptors, and detected the ERT's neuroprotection against stroke induced by middle cerebral artery occlusion (MCAO).

**Results:**

We found that the expression levels of phospho‐ERα‐S118 and estrogen receptor β (ERβ) in the striatum of OVX‐10w rats were significantly decreased and ERT's neuroprotection was abolished in the OVX‐10w rats. However, EA‐1w pretreatment could significantly recover the expression levels of phospho‐ERα‐S118 and ERβ, and also restored the neuroprotective effects of ERT in OVX‐10w rats. However, EA‐1w pretreatment could not restore the expression of estrogen receptors and ERT's neuroprotection in OVX‐20w rats.

**Conclusion:**

Taken together, our study indicates that EA may be an easy intervention that can restore the efficacy of estrogen therapy during the “critical period,” which has the potential to improve the stroke outcomes of an enormous number of long‐term post‐menopausal women. However, the time‐sensitive influences for how EA and estrogen metabolism interact with each other should be considered.

## Graphical abstract

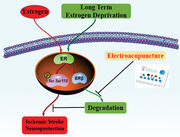

## 1 INTRODUCTION

Stroke is one of the leading causes of death and disability worldwide, and is now understood as an obviously sexually dimorphic disease (Persky et al., 2010). While men have a 33% higher incidence of stroke throughout the lifespan, women have significantly worse stroke outcomes in terms of comorbidity and mortality (Appelros et al., 2009; Lloyd‐Jones et al., 2009; Turtzo & McCullough, 2010), and a substantial number of studies have associated women's susceptibility and vulnerability to stroke to changes in the hormonal profile progression in pre‐menopausal, peri‐menopausal (ages 55−64), and post‐menopausal women. This has motivated the evaluation of hormone‐based strategies to improve stroke‐related outcomes for peri‐ and post‐menopausal women. Previous studies have established that treatment with 17β‐estradiol (E2) offers beneficial neuroprotective effects against cerebral ischemia injury in experimental rodent models (e.g. as induced by middle cerebral artery occlusion [MCAO] and by global cerebral ischemia [GCI]) (Gibson et al., 2006; Lebesgue et al., 2009; Y. L. Ma et al., 2013; Y. Ma et al., 2016). Notably, many of these studies have used ovariectomized (OVX) rats to alter estrogen metabolism, and experimental strokes have been induced at various time stages after this form of surgical menopause.

The results from clinical studies of estrogen replacement therapies (ERTs) were very challenging to interpret and appeared to be strongly influenced by age. For example, whereas it was once widely anticipated that E2 treatment would confer substantial benefits for stroke‐related outcomes in post‐menopausal women (Grady et al., 1992), an analysis of the Women's Health Initiative study surprisingly failed to observe any neuroprotective effect of E2 therapy against stroke. The study reported that E2 treatment increases the risk of ischemic stroke in generally healthy post‐menopausal women (Wassertheil‐Smoller et al., 2003). Nevertheless, a large number of studies reporting age‐related differential neuroprotective efficacy for E2 therapies led to the so‐called "critical period hypothesis" which posits that there is apparently only a limited phase of the female hormonal profile progression during which E2 therapy can confer beneficial protective effects (Suzuki et al., 2007; Zhang et al., 2011). In 2016, a large clinical study strongly supported the existence of a critical period of ERT (Hodis et al., 2016). It is now understood that the expression and molecular stability of estrogen receptor nuclear transcription factors in cells (including ERα and ERβ) can help to explain some of the observed age‐related differential neuroprotective impacts of E2 therapies (Zhang et al., 2011).

However, there have been very few studies that have explored the possibility of somehow modulating the critical period (e.g. by lengthening the duration of the effective window or enabling long‐term post‐menopausal women to regain substantially more benefit from E2 therapy). Notably, multiple studies have demonstrated that electroacupuncture (EA), a combination of traditional acupuncture with modern electrotherapy techniques, can alter endocrine metabolism and can induce significant and beneficial neuronal effects. For example, previous studies indicated that EA pretreatment at the Baihui acupoint (GV20) exerted the neuroprotective effects against cerebral ischemia (Wang et al., 2005, 2009; Xiong et al., 2003). And EA treatment could improve reproductive dysfunction by regulating estrogen metabolism (Stener‐Victorin & Wu, 2010). It has also been shown that EA affects estrogen metabolism and alters estrogen receptor levels in the hypothalamic preoptic area of OVX rats (S. Ma et al., 2011). However, there have been no reports whether EA may influence the neuroprotective effects of E2 therapies.

## 2 MATERIALS AND METHODS

### 2.1 Animals and experimental group

**FIGURE 1:**
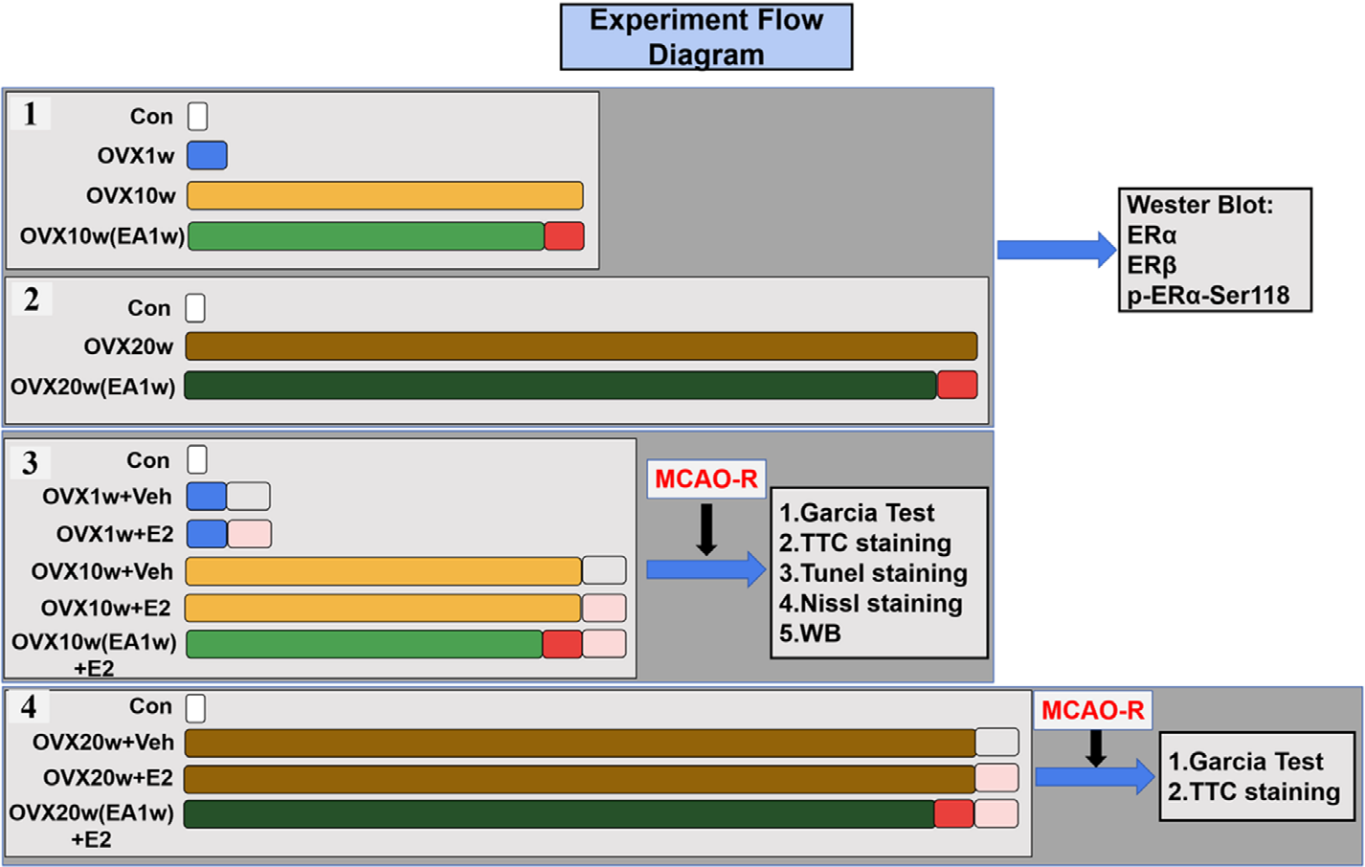
Experiment flow diagram. In Experiment 1, 36rats were divided into four groups: Con (Control), OVX1w (the mice received ovariectomy for 1 week), OVX10w (the mice received ovariectomy for 10 weeks), OVX10w(EA1w) (the mice received ovariectomy for 10 weeks and received EA treatment since the ninth week). In Experiment 2, 24 rats were divided into three groups: Control (Con), OVX20w, and OVX20w (EA1w, EA begins at OVX19w). In Experiments 3 and 4, after receiving OVX, 17β‐estrodiol (E2) or EA treatment, the rats were all subjected to MCAO‐R injury. In Experiment 3, 180 rats were divided into six groups: Con, OVX1w+Veh, OVX1w+E2, OVX10w+Veh, OVX10w+E2, and OVX10w (EA1w)+E2. In Experiment 4, 60 rats were divided into f groups: Con, OVX 20w+Veh, OVX20w+E2, and OVX20w (EA1w)+E2

Three hundred adult female Sprague–Dawley rats (weighing 250−300 g, aging 6–8 months) were obtained from the Laboratory Animal Center of Chinese PLA General Hospital in Beijing, China. There were four experiments in this study and the experiment design and group were shown in the Figure 1. In Experiment 1, 36 rats were divided into four groups: Con (Control), OVX1w (the mice received ovariectomy for 1 week), OVX10w (the mice received ovariectomy for 10 weeks), OVX10w(EA1w) (the mice received ovariectomy for 10 weeks and received EA treatment since the ninth week). In Experiment 2, 24 rats were divided into three groups: Control (Con), OVX20w, and OVX20w (EA1w, EA begins at OVX19w). In Experiments 3 and 4, after receiving OVX, 17β‐estrodiol (E2), or EA treatment, the rats were all subjected to MCAO‐R injury. In Experiment 3, 180 rats were divided into six groups: Con, OVX1w+Veh, OVX1w+E2, OVX10w+Veh, OVX10w+E2, and OVX10w (EA1w)+E2. In Experiment 4, 60 rats were divided into four groups: Con, OVX 20w+Veh, OVX20w+E2, and OVX20w (EA1w)+E2. All animals were maintained with the following standard conditions: 12 h:12 h light–dark cycle, 50–60% environmental humidity, a temperature of 25 ± 1℃ and ad lib access to food and water. We sacrificed animals for experiment by using excess chloral hydrate. The experimental procedures followed the protocols approved by the Ethics Committee for Animal Experimentation of Chinese PLA General Hospital, Beijing, China.

### 2.2 OVX and E2 replacement

OVX was performed through dorsolateral incisions as previously described (Marcondes et al., 2002). Following OVX, vaginal smears were performed to confirm the success of the OVX and the cessation of the estrous cycle. These animals then received subcutaneous injection of E2 (100 μg/kg, diluted in sesame oil solution, 2 mL) or sesame oil (Vehicle) once every other day on the back of the neck for 10 days. The dose of estrogen replacement was based on a previous study (Zhang et al., 2011).

### 2.3 Electroacupuncture pretreatment

EA treatment was performed as described in our previous study (Wang et al., 2009), with a modification to avoid the influence of anesthesia on the effect of EA treatment; we kept the rats awake during EA manipulation in a gentle immobilization apparatus designed by our laboratory (China patent application number: p201721299630.9). The acupoint “Baihui (GV 20),” which is located at the intersection of the sagittal midline and the line linking 2 rat ears, was stimulated with the intensity 4 grade and frequency of 2/15 Hz for 30 min by using an EA Instrument (Product name: Nerve and Muscle Stimulator, Type: SDZ‐V, Brand: Hwato, Manufacturer: Suzhou Medical Appliance Factory Ltd). The core temperature of all the rats was maintained at 37.0°C during EA treatment by surface heating or cooling.

### 2.4 Serum estradiol levels

The serum estradiol levels were measured to confirm the expected effects of the E2 replacement treatment (ERT). The serum estradiol levels were measured by EIA kit (Cayman Chemical).

### 2.5 Middle cerebral artery occlusion and reperfusion

The intraluminal filament model of MCAO was used to induce transient focal cerebral ischemia as in our previous study (Wang et al., 2009). Regional cerebral blood flow was measured via transcranial laser Doppler flowmetry (PeriFlux 5000, Perimed AB). Rats with >80% flow reduction during the ischemic period and >70% flow recovery within the first 10 min of reperfusion were included in the study (Figure S1 in the Supporting Information). Monitored physiological variables included rectal temperature, blood pressure, blood gas, and glucose levels (Table S1 in the Supporting Information). In each group, there were several rats that died within the reperfusion period of 72 h, which were excluded from the study.

### 2.6 Neurological scores

Neurological deficits were evaluated in a subset of the rats of each group at 72 h after reperfusion by a blinded observer based on the Garcia Test (Garcia et al., 1995).

### 2.7 Assessment of infarct volume

After neurological scoring, infarct volume was assessed via 2,3,5‐triphenyltetrazolium chloride (TTC) staining, and corrections were made for swelling, and relative infarct size was determined based on the following equation: relative infarct size = (contralateral area − ipsilateral non‐infarct area) / contralateral area according to a previous study (Wang et al., 2009).

### 2.8 Nissl staining

Nissl staining was performed to observe neuronal morphological changes within the ischemic penumbra at 72 h following reperfusion as our previous study described (Y. L. Ma et al., 2013). The experimental steps were strictly performed based on the manufacturer's manual of the Nissl staining kit (#G1430, Solarbio, China). The total number of damaged neurons in the penumbra were counted in five different fields of view for each section by an observer blinded to the treatment group manner via light microscopy at ×400 magnification (BX51; Olympus).

### 2.9 TUNEL staining

Brain sections were prepared as for the aforementioned Nissl staining, and apoptotic cells were quantified using a commercially available fluorescent terminal deoxynucleotidyl transferase nick‐end labeling (TUNEL) kit in accordance with the manufacturer's protocol (Roche Diagnostics).

### 2.10 Western blotting

We measured the protein levels of ERα and ERβ, and levels of phospho‐ERα‐Ser118 in the cortex and striatum of non‐ischemic mice; and measured Bcl‐2 and caspase‐3 protein expression in the ischemia penumbra, which were dissected as follows: in the coronal section of the brain, a sagittal section was made 2 mm apart from the middle suture, then the innermost uninjured boundary point was found, and a section from this point to the front sagittal section was made, and the area between the two sections is the ischemic penumbra (Wang et al., 2009). The ischemic penumbra in the first antibody include: rabbit anti‐ERα (1:500, Abcam), rabbit anti‐ERβ (1:500, Abcam), rabbit anti‐phospho‐ERα‐Ser118 (1:500, Abcam), rabbit anti‐Bcl‐2 (1:1000, Cell Signaling Technology), rabbit anti‐cleaved‐caspase‐3 (1:1000, Cell Signaling Technology), rabbit anti‐caspase‐3 (1:1000, Cell Signaling Technology), or mouse β‐actin (1:1000, Cell Signaling Technology) antibodies. Protein bands were visualized using a LI‐COR Odyssey System (LI‐COR Biotechnology) and were densitometrically analyzed by automated ImageJ software (NIHImage, Version 1.61).

### 2.11 Statistical analysis

Data are presented as the mean ± standard deviation, and statistical analysis was performed using SPSS version 16.0 (SPSS Inc). The neurological scores were expressed as the median with interquartile ranges and were analyzed by Kruskal–Wallis tests followed by Mann–Whitney *U* tests. Other values were analyzed using one‐way ANOVA followed by post hoc Bonferroni tests. *p* < 0.05 was considered statistically significant.

## 3 RESULTS

### 3.1 Vaginal smear results and serum estradiol levels in different groups

**FIGURE 2:**
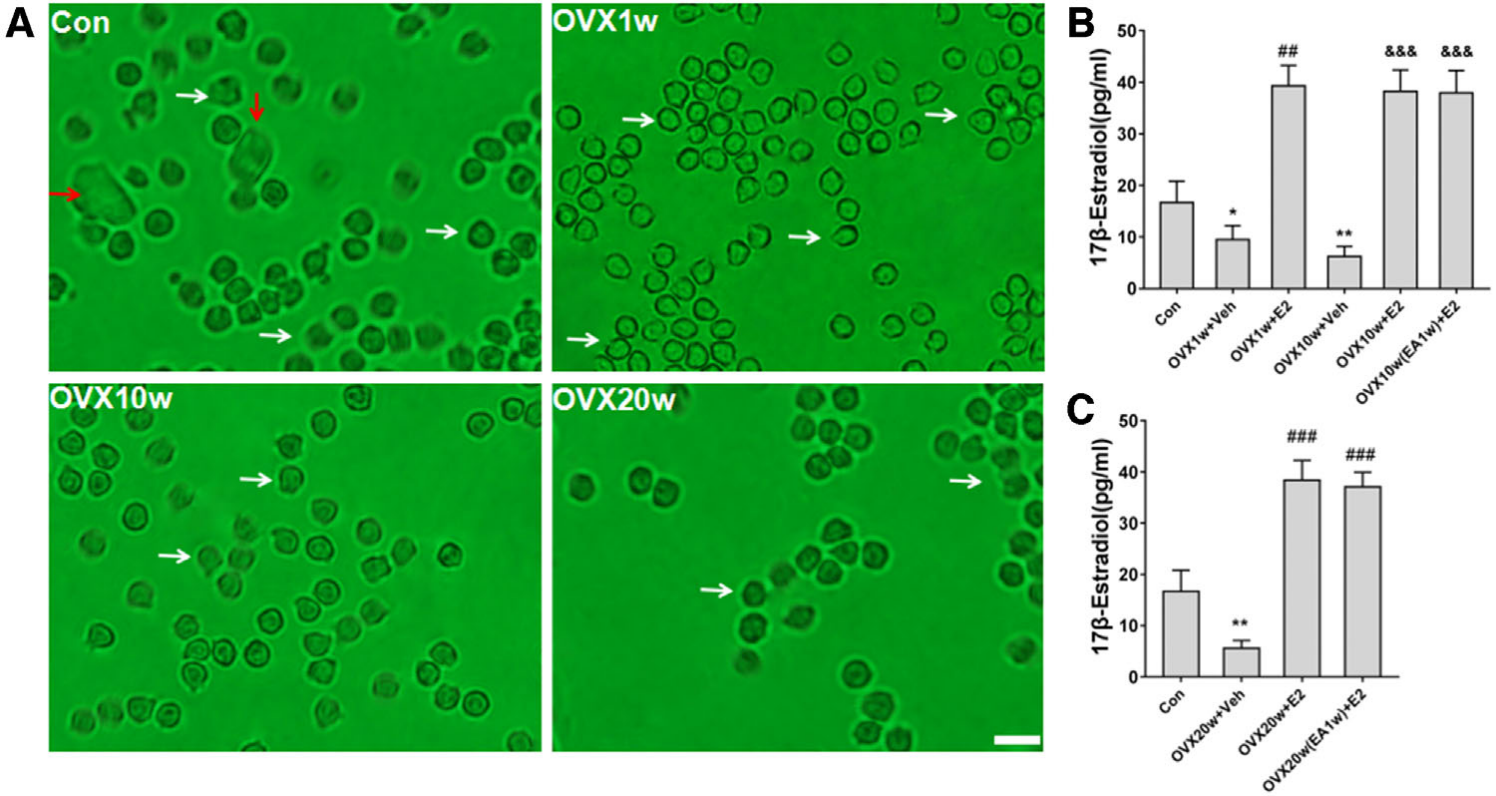
Vaginal smears and circulating estrogen levels in rats of the various treatment groups. (a) Vaginal smears of rats in the different treatment groups. Vaginal smears in the Con group consisted almost exclusively of leukocytes (white arrows) but included several cornified squamous epithelial cells (red arrows). Leukocytes were predominant in the vaginal smears of the OVX1w, OVX10w, OVX20w rats; and the number of leukocytes was significantly less than in the Con group. Bar = 20 μm. (b) Serum estrogen levels in the different treatment group. Data are expressed as mean ± SD. ^*^ *p* < .05, ^**^ *p* < .01 versus Con group; ^##^ *p* < .01 versus OVX1w+Veh group; ^&&&^ *p* < .001 versus OVX10w+Veh group; *n* = 8 per group. (c) Serum estrogen levels in the different treatment group. Data are expressed as mean ± SD. ^**^ *p* < .01 versus Con group; ^###^ *p* < .001 versus OVX20w+Veh group; *n* = 8 per group

As shown in Figure 2a, the estrous of OVX were determined via cytological evaluations of vaginal smear analysis under microscopic examination. Vaginal smears in the Con group consisted almost exclusively of leukocytes (white arrows) but included several cornified squamous epithelial cells (red arrows). Leukocytes were predominant in the vaginal smears of the OVX1w, OVX10w, OVX20w rats; and the number of leukocytes was significantly less than in the Con group. Next, the serum estradiol levels were determined to verify the effects of estrogen replacement treatment (*n* = 8). As shown in Figure 2b, the serum estradiol levels in OVX1w+Veh and OVX10w+Veh groups were significantly lower than Con group (^*^ *p* < .05, ^**^ *p* < .01). The serum estradiol levels in OVX1w+E2 was significantly higher than OVX1w+Veh group (^##^ *p* < .01). The serum estradiol levels in both OVX10w+E2 group and OVX10w(EA1w)+E2 group were significantly higher than OVX10w+Veh group (^&&&^ *p* < .001). As shown in Figure 2c, the serum estradiol levels in OVX20w+Veh group were significantly lower than Con group (^**^ *p* < .01). The serum estradiol levels in both OVX20w+E2 group and OVX20w(EA1w)+E2 group were significantly higher than OVX20w+Veh group (^###^ *p* < .001).

### 3.2 Electroacupuncture restores the expression of estrogen receptor in the striatum but not in the cortex of long‐term OVX rats

**FIGURE 3:**
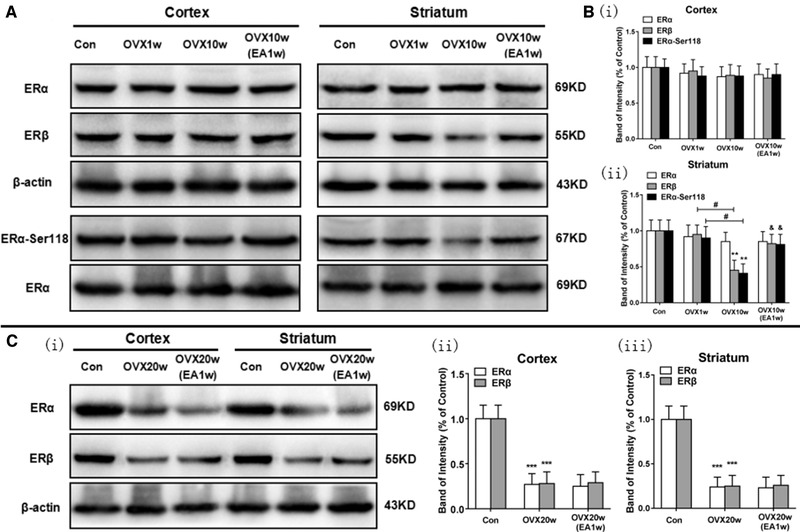
EA treatment restored the accumulation of ERβ and phosphor‐ERα‐Ser118 in the striatum of OVX‐10w rats. (a) Representative photographs of cropped gels and immunoblots showing the accumulation of ERα and ERβ proteins as well as phosphor‐ERα‐Serine118 in the cortex and striatum for the various treatment groups. (b) Graph of the quantitation for the signals in the cortex (i) and striatum (ii) as shown in (a). Data are expressed as mean ± SD. ^**^ *p* < .01 versus Con group; ^#^ *p* < .05, ^##^ *p* < .01 versus OVX‐1w group; ^&^ *p* < .05 versus OVX‐10w group; *n* = 6 per group. (d) (i) Representative photographs of cropped gels and blots showing the expression of the ERα and ERβ protein in the cortex and striatum in the different groups. (ii) Graph of the ERα and ERβ protein in the cortex, and (iii) striatum in the different group. Data are expressed as mean ± SD. ^***^ *p* < .001 versus Con group; *n* = 6 per group

We used immunoblotting to examine the levels of ERα, ERβ, and phospho‐ERα‐S118 in both the cortex and striatum brain regions in different groups (*n* = 6). We found that in the cortex, there are no significant differences in the levels of ERα, ERβ, and phospho‐ERα‐S118 in the Con, OVX1w, OVX10w, and OVX10w(EA1w) group as shown in Figure 3a,b(i). In the striatum, there are no significant differences in the levels of ERα in the Con, OVX1w, OVX10w, and OVX10w(EA1w) groups as shown in Figure 3a,b(ii); and there are no significant differences about the levels of ERβ and phospho‐ERα‐S118 between the Con and OVX1w group as shown in Figure 3a,b(ii); however, the levels of ERβ and phospho‐ERα‐S118 in the OVX10w group were both significantly decreased (^**^ *p* < .01 vs. Con group, ^#^ *p* < .05 vs. OVX1w group); but EA1w treatment which began at OVX9w (OVX10w(EA1w) group) could significantly restore the levels of ERβ and phospho‐ERα‐S118 (^&^ *p* < .05 vs. OVX10w group) as shown in Figure 3a,b(ii).

Then we detected the levels of ERα and ERβ in both the cortex and striatum brain regions in OVX20w and OVX20w(EA1w) group as shown in Figure 3c (*n* = 6). We found that the levels of ERα and ERβ in both the cortex and striatum brain regions in OVX20w group were significantly decreased compared with the Con group (^***^ *p* < .001); but EA1w treatment which began at OVX19w in OVX20w(EA1w)+E2 group could not restore the levels of ERα and ERβ.

### 3.3 Electroacupuncture restores the neuroprotective effects of estrogen therapy following OVX surgery

**FIGURE 4:**
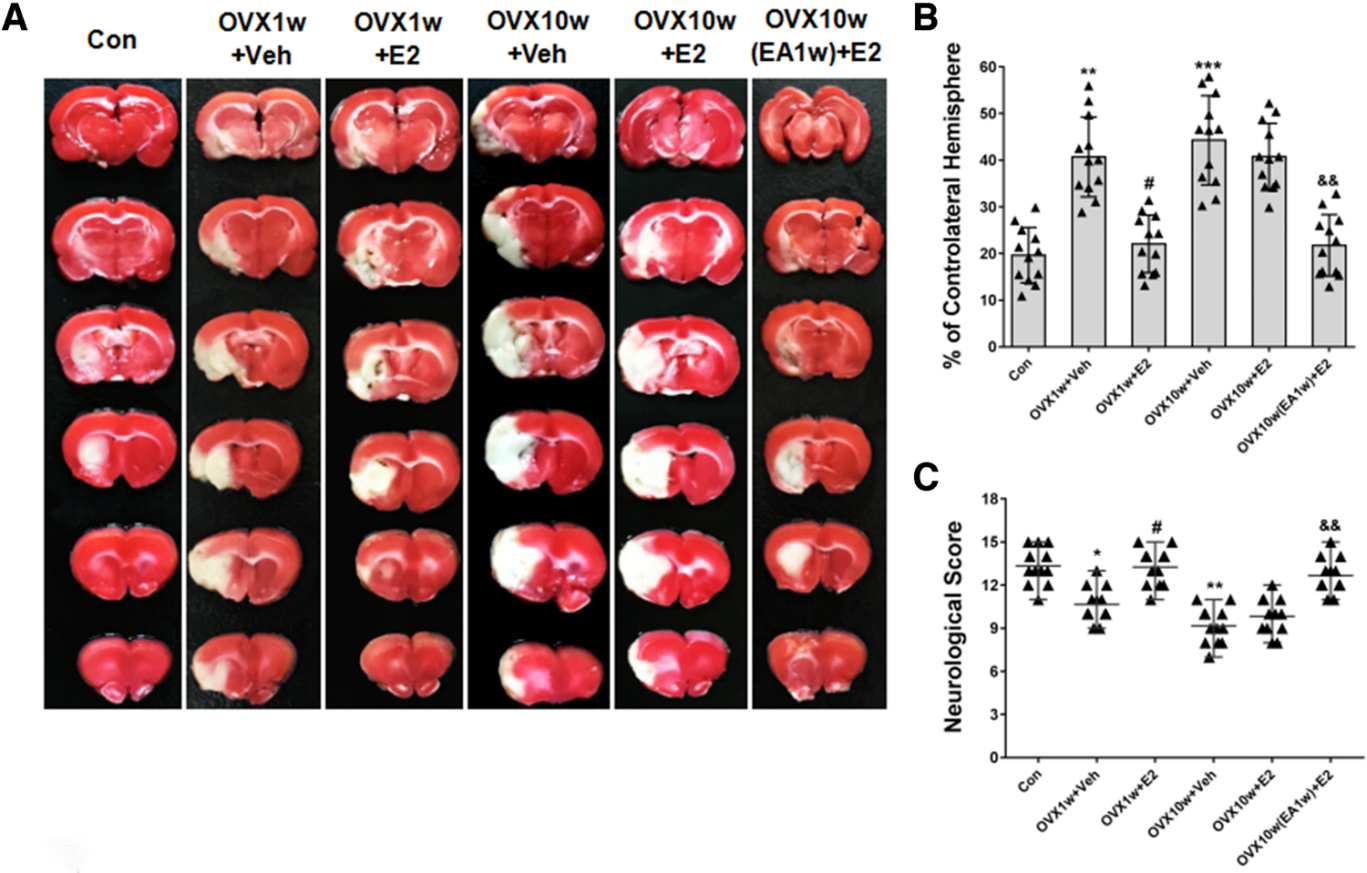
EA treatment restored the neuroprotective effect of E2 against cerebral ischemia/reperfusion injury in OVX‐10w rats. (a) TTC staining after ischemia‐reperfusion for the various treatment groups. Representative photographs showing infarct volumes in various treatment groups following cerebral ischemia/reperfusion. (b) Graph of the infarct volume in rats undergoing cerebral ischemia/reperfusion in the different treatment groups. Data are expressed as mean ± SD. ^**^ *p* < .01, ^***^ *p* < .001 versus Con group; ^#^ *p* < .05 versus OVX1w+Veh group; ^&&^ *p* < .01 versus OVX10w+E2 group; *n* = 12 per group. (c) Neurological scores after cerebral ischemia/reperfusion in the different groups. The Garcia Test was applied to evaluate the neurological deficits in the rats after cerebral ischemia/reperfusion injury in the different groups. Data are expressed as median ± interquartile range. ^*^ *p* < .05, ^**^ *p* < .01 versus Con group; ^#^ *p* < .05 versus OVX1w+Veh group; ^&^ *p* < .05 versus OVX10w+E2 group; *n* = 12 per group

We next used MCAO model to induce transient focal cerebral ischemia 2 days after the cessation of the E2 (or vehicle) treatments at both 1 week and 10 week post OVX stages (*n* = 12). As shown in Figure 4a,b, the infarct volume was larger in OVX1w+Veh group than that of Con group (^**^ *p* < .01), which represented much worse neurological situation. However, E2 treatment (OVX1w+E2 group) significantly decreased the infarct volume compared with that of OVX1w+Veh group (^#^ *p* < .05). At 10 week post OVX stage, the OVX10w+Veh group rats had significantly larger infarct volumes than the Con group rats (Figure 4a,b, ^***^ *p* < .001). Moreover, these OVX10w+Veh group rats had slightly but not significantly larger infarct volumes than the OVX10w+E2 rats (Figure 4a,b). Excitingly, EA treatment (30 min/day, 1 week) starting at 9 week post OVX followed by E2 treatment in OVX10w(EA1w)+E2 group rats led to a significant reduction in infarct volumes as compared to the OVX10w+E2 rats (Figure 4a,b, ^&&^ *p* < .01). The infarct volume of these OVX10w(EA1w)+E2 group rats did not differ from OVX1w+E2 group and Con group rats (Figure 4a,b). This is an important finding in the context of the “critical period hypothesis,” as our observations suggested that EA treatment, even when applied relatively longer after OVX, could apparently restore the efficacy of E2 therapy to such an extent that it did not differ from rats with functional (estrogen‐producing) ovaries. Extending the potential purport of this intriguing observation, we observed precisely the same trends for each of the groups when we assessed neurological scores. As shown in Figure 4c, compared with the Con group, the OVX1w+Veh group and OVX10w+Veh group had significantly lower neurological scores, indicating a worse neurological condition (^*^ *p* < .05, ^**^ *p* < .01). However, the neurological scores of the rats in the OVX1w+E2 group were dramatically increased compared with that of the OVX1w+Veh group (^#^ *p* < .05). And no significant neurological score difference was observed between the OVX10w+Veh group and OVX10w+E2 groups. Interestingly, EA treatment in OVX10w(EA1w)+E2 group rats significantly increased the neurological scores compared with that of OVX10w+E2 group (^&&^ *p* < .01).

**FIGURE 5:**
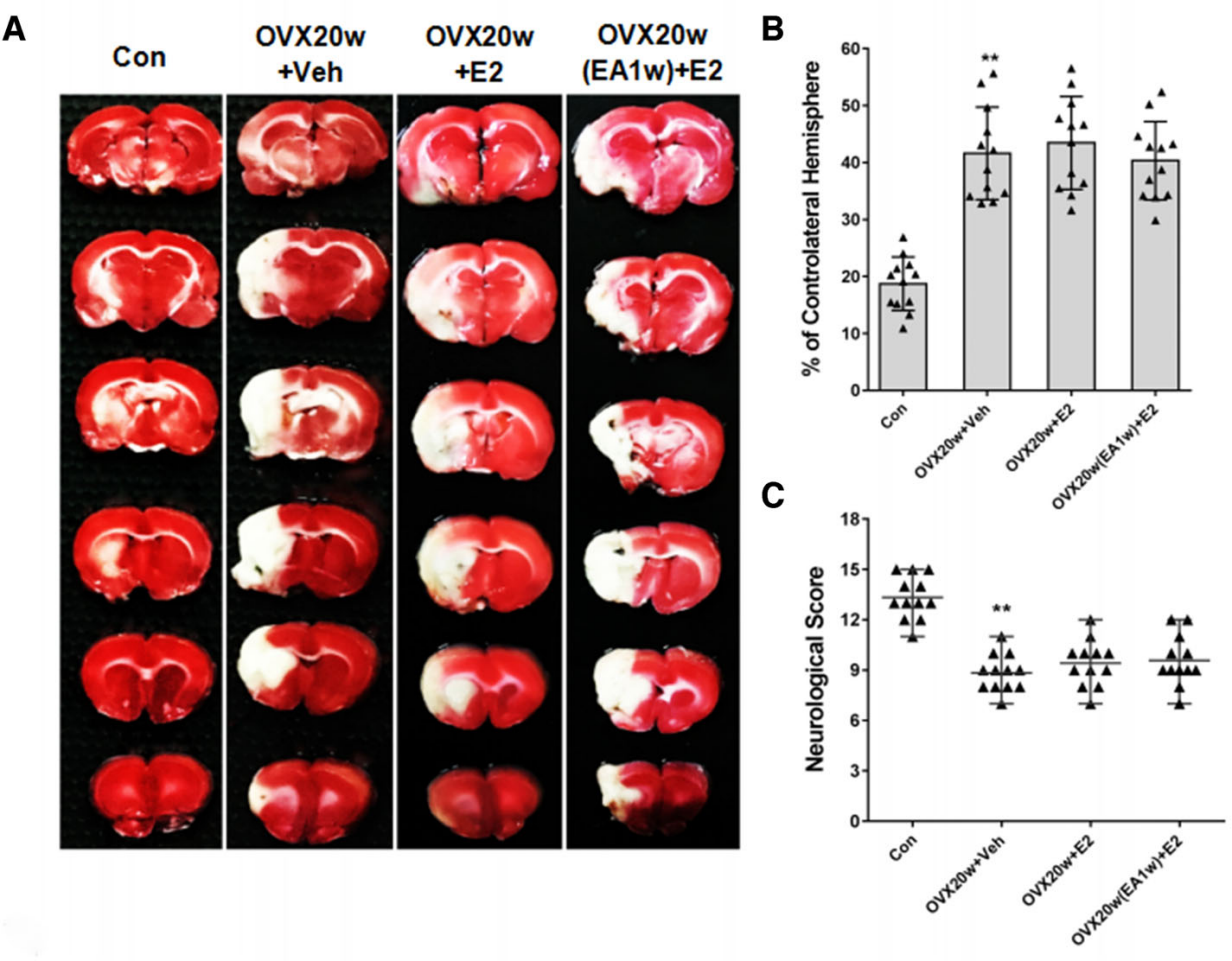
**E**A treatment did not restore the neuroprotective effect of E2 against cerebral ischemia/reperfusion injury in OVX‐20w rats. (a) TTC staining after cerebral ischemia/reperfusion in the various treatment groups. Representative photographs showing infarct volumes in the different groups following cerebral ischemia/reperfusion. (b) Infarct volumes in rats after cerebral ischemia/reperfusion in the different treatment groups. Data are expressed as mean ± SD. ^**^ *p* < .01 versus Con group; *n* = 12 per group. (c) Neurological scores after cerebral ischemia/reperfusion in the different groups. The Garcia Test was applied to determine neurological deficits in the rats after cerebral ischemia/reperfusion injury in the different group. Data are expressed as median ± interquartile range. ^**^ *p* < .01 versus Con group; *n* = 12 per group

Encouraged by the very promising neuroprotective effects at relatively late stages post OVX that resulted from EA treatment, we conducted similar experiments with rats at 20 week post OVX stage (Experiment 4) (*n* = 12). Similarly, as shown in Figure 5, this finding was in agreement with our aforementioned results (OVX10w+Veh group vs. Con group) showing that the rats in the OVX20w+Veh group had larger infarct volume with worse neurological scores compared with that of the Con group (^**^ *p* < .01). However, in sharp contrast to the results observed with the OVX10w rats, there was obviously a time‐sensitive window within which EA treatment could confer its effects in boosting the efficacy of E2 treatment. We did not observe significant differences in either infarct volumes or neurological scores among OVX20w+Veh, OVX20w+E2 and OVX20w(EA1w)+E2 group rats (Figure 5). Notably, this finding was in agreement with our aforementioned results showing time‐sensitive (OVX10w vs. OVX20w) responses to EA treatment for the estrogen receptor protein levels (Figure 3).

### 3.4 Electroacupuncture reduces the extent of cellular and biochemical events associated with poor post stroke outcomes

**FIGURE 6:**
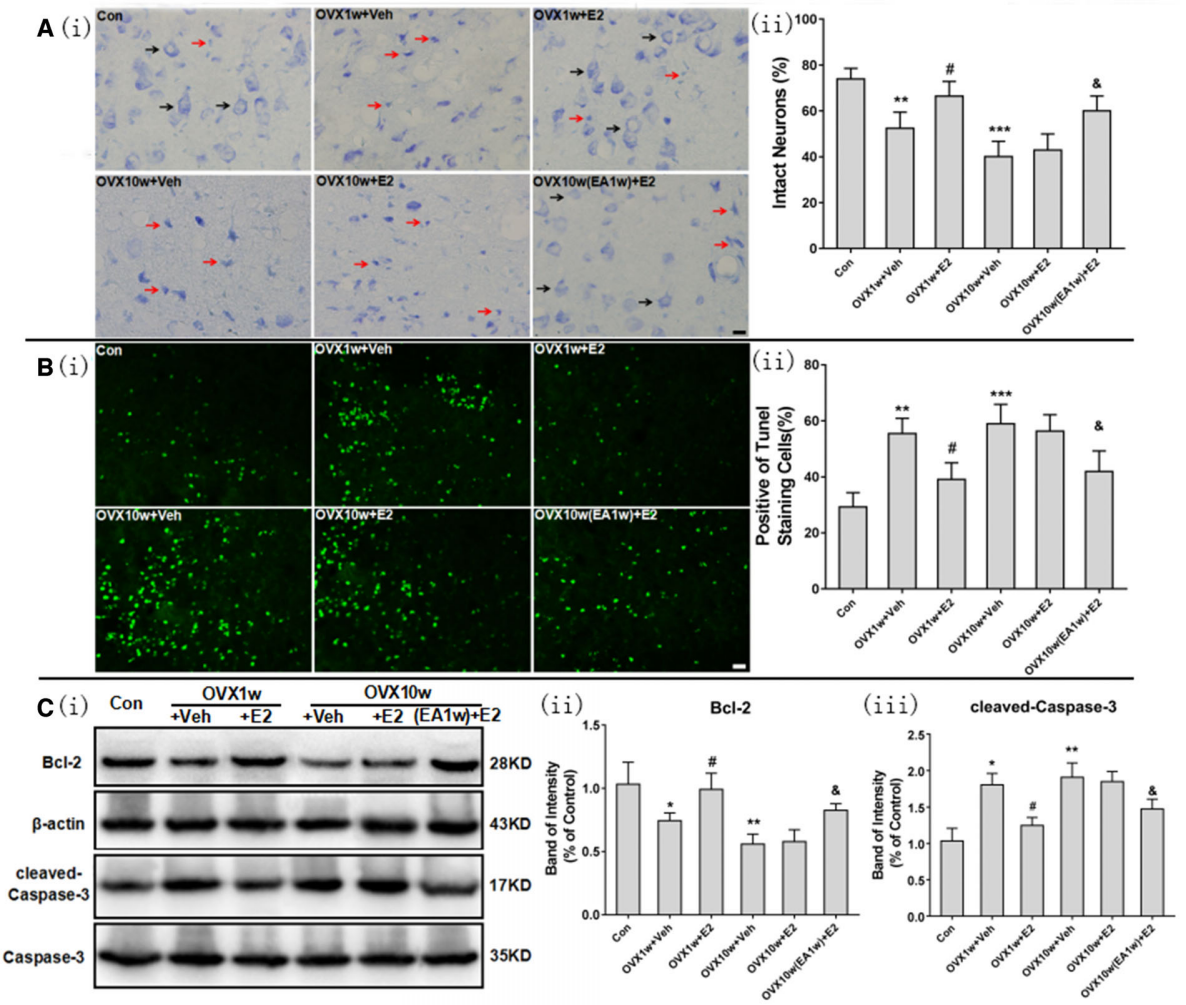
EA treatment restored the neuroprotective effect of E2 treatment against neuron injury and cell apoptosis in ischemic penumbras after cerebral ischemia/reperfusion in rats in OVX‐10w rats. (a) Nissl staining of morphological changes to neurons in ischemic penumbras after cerebral ischemia/reperfusion. (i) Representative photographs showing morphological changes to neurons in ischemic penumbras after cerebral ischemia/reperfusion. Black arrows represented the intact neurons and red arrows represented the injured neurons. Bar = 10 μm. (ii) Graph of the number of intact neurons in the ischemic penumbras of the different groups. Data are expressed as mean ± SD. ^**^ *p* < .01, ^***^ *p* < .001 versus Con group; ^#^ *p* < .05 versus OVX1w+Veh group; ^&^ *p* < .05 versus OVX10w+E2 group; *n* = 6 per group. (b) TUNEL staining indicating cell apoptosis in ischemic penumbras following cerebral ischemia/reperfusion. (i) Representative photographs showing neuronal apoptosis in ischemic penumbras. Bar = 10 μm. (ii) Graph of the extent of apoptosis in the ischemic penumbras of the different groups. Data are expressed as mean ± SD. ^**^ *p* < .01, ^***^ *p* < .001 versus Con group; ^#^ *p* < .05 versus OVX1w+Veh group; ^&^ *p* < .05 versus OVX10w+E2 group; *n* = 6 per group. (c) Accumulation of the Bcl‐2 and cleaved‐caspase‐3 proteins in ischemic penumbras following cerebral ischemia/reperfusion. (i) Representative photographs of cropped gels and immunoblots showing the expression of the Bcl‐2 and cleaved‐caspase‐3 proteins in the different treatment groups. (ii, iii) Quantitation of the signals for the Bcl‐2 and cleaved‐caspase‐3 proteins in the different treatment groups. Data are expressed as mean ± SD. ^*^ *p* < .05, ^**^ *p* < .01 versus Con group; ^#^ *p* < .05 versus OVX1w+Veh group; ^&^ *p* < .05 versus OVX10w+E2 group; *n* = 6 per group

Driven by our observations of the apparent substantial neuroprotective effects of EA treatment for OVX rats, we next examined whether or not known cellular or other biomolecular contributors to neurodegeneration and poor post‐stroke outcomes were differentially affected in the various treatment groups (*n* = 6). Specifically, Nissl staining revealed that there was obvious and significant decrease in the number of intact neurons in the penumbra of OVX1w+Veh group rats and OVX10w+Veh rats compared with Con group rats (Figure 6a, ^**^ *p* < .01, ^***^ *p* < .01); E2 treatment started at OVX1w significantly increased the numbers of intact neurons (^#^ *p* < .05 vs. OVX1w+Veh group), but E2 treatment started at OVX10w did not significantly increase the number of intact neurons compared with OVX10w+Veh group rats; EA treatment started at OVX9w followed by E2 treatment significantly increased the number of intact neurons (Figure 6a, ^&^ *p* < .05 vs. OVX10w+E2 group).

Consistent with the results of Nissl staining, TUNEL staining showed that the extent of cells undergoing apoptosis in the penumbra were substantially and significantly higher in OVX1w+Veh group rats and OVX10w+Veh rats compared with Con group rats (Figure 6b, ^**^ *p* < .01, ^***^ *p* < .01); E2 treatment started at OVX1w significantly decreased the number of apoptosis cells (Figure 6b, ^#^ *p* < .05 vs. OVX1w+Veh group), but E2 treatment started at OVX10w did not significantly decrease the number of apoptosis cells compared with OVX10w+Veh group rats; however, EA treatment started at OVX9w followed by E2 treatment significantly decreased the number of intact neurons (Figure 6b, ^&^ *p* < .05 vs. OVX10w+E2 group).

Bcl‐2 is an antiapoptotic protein, and cleaved‐caspase‐3 is the key enzyme related to cell apoptosis, which plays a pivotal role in the process. We found that ovariectomy significantly decreased the expression of Bcl‐2 and increased the expression of cleaved‐caspase‐3 in the penumbra following ischemic stroke injury (Figure 6c, ^*^ *p* < .05, ^**^ *p* < .01 vs. Con group). E2 treatment in OVX1w+E2 group significantly increased the Bcl‐2 expression and decreased the cleaved‐caspase‐3 expression compared to that of OVX1w+Veh group (^#^ *p* < .05). And EA treatment profoundly increased Bcl‐2 levels and reduced the cleaved‐caspase‐3 levels (^&^ *p* < .05 vs. OVX10w+E2 group). The trends of apoptosis‐related proteins expression, including Bcl‐2 and Caspase‐3, were consistent with both the cell biological and infarct volume results, further confirming the effect of the EA treatment in restoring the neuroprotective efficacy of the E2 treatment.

## 4 DISCUSSION

In the present study, we used an OVX rat model to test if EA could help to restore the neuroprotective effects of E2 treatment after a long‐term surgical menopause. Our results revealed that EA does significantly affect the levels of estrogen receptor proteins in the stratum region of OVX rat brains. EA treatment dramatically recovered the neuroprotective efficacy of E2 treatment in rats at 10 week post OVX. As this type of EA‐potentiated improvement was not observed in rats at 20 weeks post OVX, there are clearly time‐sensitive influences on how EA and estrogen metabolism interact to confer neuroprotective effects, which is perhaps similar to what is known about the differential benefits of E2 in human females at the peri‐ versus post‐menopause phases of the hormonal progression.

Our findings modify the current understanding of E2 therapy in OVX rats in several ways. ERT exerts its physiological effects mainly via facilitating the binding of estrogen to classic estrogen receptors (ER) α or β. In response to estradiol binding, ERα needs to be phosphorylated, predominately on Serine‐118, while phosphorylation of ERβ has not been examined in detail (Anbalagan & Rowan, 2015; Lannigan, 2003 ). Previous studies have indicated that ERα may be more relating to injury‐induced E2‐mediated protection, whereas ERβ may play an important role in the basal neuroprotection of estrogen (Scott et al., 2012). A previous study by Zhang et al. (2011) found that E2 neuroprotection against GCI injury was lost in OVX‐10w rats, which was associated with a significant decrease in the cellular populations of the ERα protein in the hippocampus due to the ubiquitination/degradation induced by Chip interaction. And GCI is generally considered as an ischemia model to mimic cerebral ischemia induced by cardiac arrest.

In the present study, we used an MCAO model, which is thought to better mimic clinical ischemic stroke, and which typically causes injury to the cortex and striatum regions of the brain, with especially pronounced effects on the striatum (Carmichael, 2005; Liu et al., 2009). Similar to the Zhang et al. (2011) study, we observed here that the neuroprotective effects of estrogen (in our case, against MCAO injury) were not evident in OVX‐10w rats. Importantly, we observed a significant decrease in ERβ and phospho‐ERα‐Ser118 protein levels in the striatum of the OVX‐10w rats rather in the cortex of the OVX‐10w rats, which potentially help to explain the loss of efficacy of E2. The OVX‐20w rats of our study had significantly decreased accumulation of ERα and ERβ both in the cortex and striatum regions, which was similar to the previously reported finding of significantly decreased levels of both ERα and ERβ in 24‐month‐old non‐OVX rats (nearly 12 months after typical menopause in rats) (Zhang et al., 2011). These differences may be due to the different sensitivity to estrogen regulation between cortex and striatum. After E2 or ER agonist administration, more kinds of neurotransmitters changed in striatum compared with cortex (Long et al., 2019), which indicated the high sensitivity to E2 in the striatum. Therefore, we inferred that 10 weeks estrogen deprivation led to ER degradation in high estrogen‐sensitive region, striatum, but not in cortex; whereas, longer estrogen deprivation for 20 weeks led to ER degradation both in cortex and striatum, regardless of the E2 sensitivity.

In this study, we first found that EA treatment increases the expression of the ERβ protein and the levels of phospho‐ERα‐Ser118 in OVX‐10w rats. A previous study reported that ERα and ERβ levels were increased in the hypothalamus in OVX‐4w rats, and animals which received EA treatment for 3 days at 4 week post OVX did not show similar increases in these proteins (S. Ma et al., 2011). In the present study, we found that ERβ levels were increased in the striatum, which is different from the previous report. The hypothalamus is known to be a key region for regulating reproductive function in females and is understood as the most susceptible brain region to estrogen loss, and considering that their study treated the acupoints “Guanyuan” (CV4), “Zhongji” (CV3), “Zigong” (EXTRA22), and “Sanyinjiao” (SP6), which are associated with reproductive rather than neurological function. Therefore, it is obvious that additional experiments would be needed to untangle these apparently opposite trends for estrogen receptor accumulation in these EA‐treated brain regions and the underlying mechanisms.

It is also notable that the present study is the first to report the existence of an apparent critical period for estrogen neuroprotection against ischemic stroke when using a MCAO model in OVX rats. We are not aware of any other reports that EA treatment can alter ERβ or phospho‐ERα‐Ser118 levels in the striatum regions of OVX rats, a finding which indicates a potential mechanism for the observed ability of EA to recover the critical period for E2 therapy in long‐term OVX animals.

We propose the term “peri‐critical period” to describe this critical‐period‐modulating concept for this experimental system. A previous study found that donepezil treatment could restore the ability of estrogen to improve the cognitive performance in aged rats by increasing cholinergic function, which confirmed the role of estrogen in the critical period hypothesis (Gibbs et al., 2009). Future studies which add additional treatments in the window between the 10 week and 20 week post OVX will add temporal resolution to our understanding of this peri‐critical period and should facilitate the design of both the EA and E2 experiments which should ultimately facilitate the full elucidation of the biochemical and electrophysiological events underlying our observations. Considering that simple but powerful hormone replacement therapies can substantially improve stroke‐related outcomes in a vast number of women, the practical impacts of our finding that EA may be an easy way to recover the critical period could be quite large.

## FUNDING INFORMATION

This study was funded by National Natural Science Foundation of China (No. 82101427, 82171464, 81801138), Beijing Municipal Science & Technology Commission (No. 1811000017180022), The National Key Research and Development Program of China (2019YFC0121703) and Chinese PLA General Hospital Military Medicine Innovation Research Project (CX19030).

## CONFLICT OF INTEREST

The authors declare no conflict of interest.

### PEER REVIEW

The peer review history for this article is available at https://publons.com/publon/10.1002/brb3.2316

## Data Availability

The data that support the findings of the current study are available from the corresponding author on reasonable request.
