# Supplementary material for: Electroacupuncture reactivates estrogen receptors to restore the neuroprotective effect of estrogen against cerebral ischemic stroke in long‐term ovariectomized rats
Source: Brain Behav. 2021 Sep 2;11(10):e2316. doi: 10.1002/brb3.2316 (PMC8553307; doi:10.1002/brb3.2316)
Supplement: Supplementary file 1 [file BRB3-11-e2316-s001.doc]

**Figure S1**

**
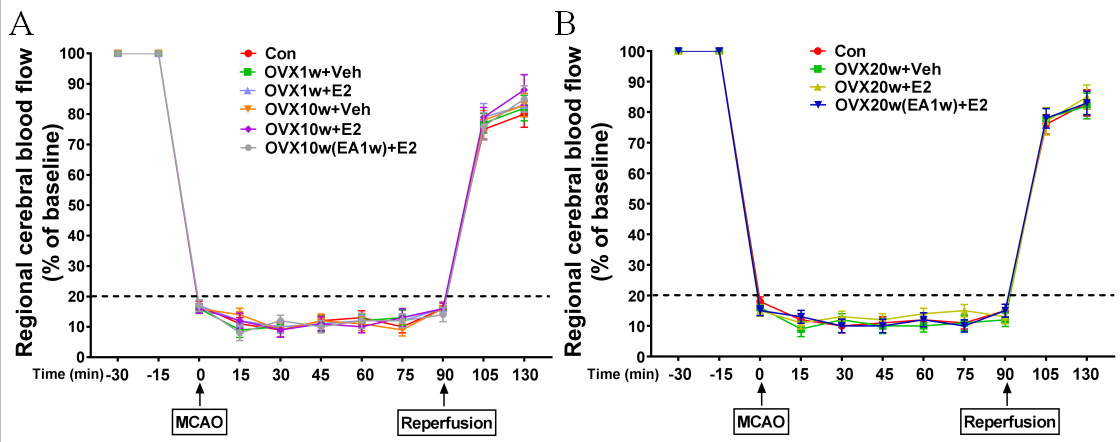
**

**Supplementary Figure 2. Regional cerebral blood flow in ischemic hemispheres of rats during MCAO surgery in the different groups.** Data are expressed as mean ± SD. N>30 per group.

**Table S1**

| Group | | MABP  (mmHg) | Temp  (℃) | Glu  （dl/ml） | Hct  (%) | pH | pO_2_  (mmHg) | pCO2  (mmHg) |
| --- | --- | --- | --- | --- | --- | --- | --- | --- |
| Con | pre | 73±2 | 37±0.2 | 192±13 | 35±3 | 7.4±0.1 | 132±4 | 46±5 |
|  | during | 69±1 | 37±0.1 | 175±10 | 31±4 | 7.4±0.1 | 148±5 | 61±6 |
|  | post | 67±2 | 37±0.2 | 171±11 | 27±2 | 7.4±0.1 | 152±7 | 56±8 |
| OVX1w+Veh | pre | 72±2 | 37±0.2 | 195±14 | 36±4 | 7.4±0.1 | 136±7 | 49±8 |
|  | during | 70±1 | 37±0.2 | 170±10 | 33±3 | 7.4±0.1 | 149±4 | 63±10 |
|  | post | 69±2 | 37±0.3 | 168±12 | 28±4 | 7.4±0.1 | 153±7 | 57±11 |
| OVX1w+E2 | pre | 72±3 | 37±0.3 | 193±15 | 35±5 | 7.4±0.1 | 139±8 | 46±6 |
|  | during | 70±1 | 37±0.3 | 170±12 | 32±3 | 7.4±0.1 | 147±5 | 65±8 |
|  | post | 67±3 | 37±0.2 | 169±11 | 26±4 | 7.4±0.1 | 155±6 | 55±11 |
| OVX10w+Veh | pre | 71±2 | 37±0.2 | 193±14 | 36±4 | 7.4±0.1 | 137±6 | 48±9 |
|  | during | 65±1 | 37±0.2 | 174±10 | 34±2 | 7.4±0.1 | 142±4 | 57±8 |
|  | post | 68±2 | 37±0.3 | 164±12 | 28±3 | 7.4±0.1 | 136±4 | 53±6 |
| OVX10w+E2 | pre | 71±3 | 37±0.3 | 190±15 | 35±4 | 7.4±0.1 | 140±5 | 48±5 |
|  | during | 64±1 | 37±0.3 | 176±12 | 32±3 | 7.4±0.1 | 142±4 | 56±7 |
|  | post | 65±3 | 37±0.2 | 168±11 | 26±4 | 7.4±0.1 | 150±4 | 56±8 |
| OVX10w(EA1w)+E2 | pre | 71±3 | 37±0.3 | 184±15 | 35±4 | 7.4±0.1 | 138±6 | 48±8 |
|  | during | 68±1 | 37±0.3 | 169±12 | 31±2 | 7.4±0.1 | 145±4 | 59±7 |
|  | post | 66±3 | 37±0.2 | 165±11 | 27±3 | 7.4±0.1 | 150±5 | 51±7 |

MABP: mean arterial blood pressure; Temp: rectal temperature; Glu: Glucose; Hct: Hematocrit.
